# Research on the evolutionary patterns and control of surrounding rock superimposed stress field local area loading in double-layer Island face main roadway

**DOI:** 10.1038/s41598-024-62466-5

**Published:** 2024-08-07

**Authors:** Dongdong Chen, Zijian Li, Zhifeng Zhang, Fulian He, Fuxing Xie, Hao Pan, Zhiqiang Wang

**Affiliations:** 1 School of Energy and Mining Engineering, China University of Mining and Technology-Beijing, Beijing, 100083 China; 2 Beijing China Coal Mine Engineering Co., Ltd., Beijing, 100013 China

**Keywords:** Double-layer island working surface, Main roadway coal pillar width, Failure mode of surrounding rock, Peak deviatoric stress zone, Asymmetric key area collaborative support, Energy harvesting, Coal

## Abstract

Double-layer island working face main roadway coal pillars are affected by complex mining stress superposition, when different coal pillar width combinations, the surrounding rock stress field will produce different degrees of regional loading increase effect; the study of the surrounding rock stress field regional superposition loading increase law is meaningful to explaining the failure mode of the roadway and determining the critical control area. This study combines numerical simulation with on-site monitoring and other methods and draws the following conclusions: The superimposed loading increase law (“decreasing” → “increasing”) of the abutment pressure and deviatoric stress in the lower coal seam of the double-layer island working face during the mining; the type of the principal stress deflection in the advance working face region; and by obtaining the three types of development morphology of the deviatoric stress peak zone of the roadway and its corresponding nine evolution modes (one type of circular tube → four types of inverse hyperbolic body → four types of hyperbolic body) in the double-layered island working face mining. Indicated the critical reinforcement area corresponding to the main roadway when at different combinations of coal pillar widths; determined the main track roadway protective coal pillars width for 40 m and the shape of the roadway peak deviatoric stress zone is the inverse class hyperbolic body mode; according to the evolution mode of the peak deviatoric stress zone, determined the synergistic failure control program for the asymmetric critical zone of the roadway surrounding rock which is a targeted scientific support method; after the feedback of on-site monitoring and, the support program is reasonable and effective.

## Introduction

China is a major coal-producing country and a significant coal-demanding country, China’s energy sector remains extremely dependent on coal^1^. With continuous mining, the conflict between the energy industry’s steady demand for coal production and the dwindling domestic reserves of mineable coal becomes increasingly apparent. In addition to optimizing the coal mining process and increasing the number of mineable sections, reducing the waste of resources in mineable areas and improving the coal recovery rate are also effective responses to ensure the output and supply of coal under limited reserves^2–4^. Nearby coal seams account for a large proportion of the mined coal reserves in China^5^. With continuous mining, the emergence of multi-layer, multi-side mining island working face is inevitable; the study of the geological conditions under the law of the emergence of mine pressure and the surrounding rock control method is to ensure the coal industry sustainable development.

With the development of the coal industry, the disturbance problem of the mine-out area and coal pillar on the underlying coal seam has become more and more prominent. In order to complete the mining of the underlying coal seam with both safety and a high resource recovery rate, it is essential to have a correct understanding of the destruction mechanism of the surrounding rock under the environment of this kind of complex stress superposition, to guide the formulation of reasonable support program. Scholars^2,6–13^ made plenty of explorations and discussions on the manifestation and control of mining pressure in the mining area under close-distance seams conditions. Pan et al.^1^, Chen et al.^4^, Cao et al.^14^, Qin et al.^15^ and Wang et al.^16^ investigated the structural transport and fissure development characteristics of the overlying rock strata during proximity multi-seam mining and discussed the effect of thelower seam mine-out on the stability of the upper seam. Li et al.^17^, Sui et al.^18^ and He et al.^19^ found that the mine-out of the upper coal seam causes the decompression effect that occurs in the lower coal seam but also results in a more intense dynamic pressure manifestation in the local area of the lower coal seam. Some scholars explore the essence of roadway destabilization from the intuitive perspective of the width of the coal pillar. Roadway safety and stability are directly correlated with the coal pillar’s width. The degree of coal seam’s stress loading in the advanced working face area will have intuitive evolution with different widths of coal pillars^11,20–23^. While deeply understanding the destruction mechanism of surrounding rock, scholars have also widely discussed the roadway instability's response program, such as applying drilling pressure relief technology to prevent and control high hazardous mining pressure problems such as impact ground pressure^5,11–13,23–25^.

Scholars have carried out abundant research on the above content. However, there is no exploration of the influence of complex mining stress superimposed on the main roadway surrounding rock under the double-layer island working face, and there is a lack of in-depth understanding of the evolution mode of deformation and failure of the main roadway under such working conditions. In applying the original theory and method to guide the deformation control, workers find it difficult to do “the right medicine”, which directly affects the safety and efficiency of the mine mining. Therefore, it is essential to adopt reasonable research indexes and explore the damage mode of the surrounding rock and its support under the double-layer island working face geological conditions.

This paper adopts the abutment pressure and deviatoric stress as indexes and studies the regional loading law of abutment pressure and the superimposed deflection law of the principle stress direction of the advanced working face area, as affected by the superposition of complex mining stress during the mining of the double-layer island face; and the deviatoric loading law of the deviatoric stress field of the coal seam’s advance working face area when at different mining steps. According to the evolution law of the peak deviatoric stress zone, proposed three types of development patterns and their corresponding nine types of evolution modes that the peak deviatoric stress zone may present under different degrees of superposition. Determined a reasonable main roadway coal pillar width and the critical area of the surrounding rock support of the double-layer island working face, combined with the principal stress direction to form a critical area synergistic control program that can effectively control the asymmetric failure of the surrounding rock, and the on-site monitoring results show that the control method is effective. The conclusions of this paper can provide valuable reference and guidance for determining the reasonable width of the coal pillar and effective control of deformation damage of the main roadway under similar geological conditions.

## Engineering geological characteristics of double-layer isolated Island working face

**Figure 1:**
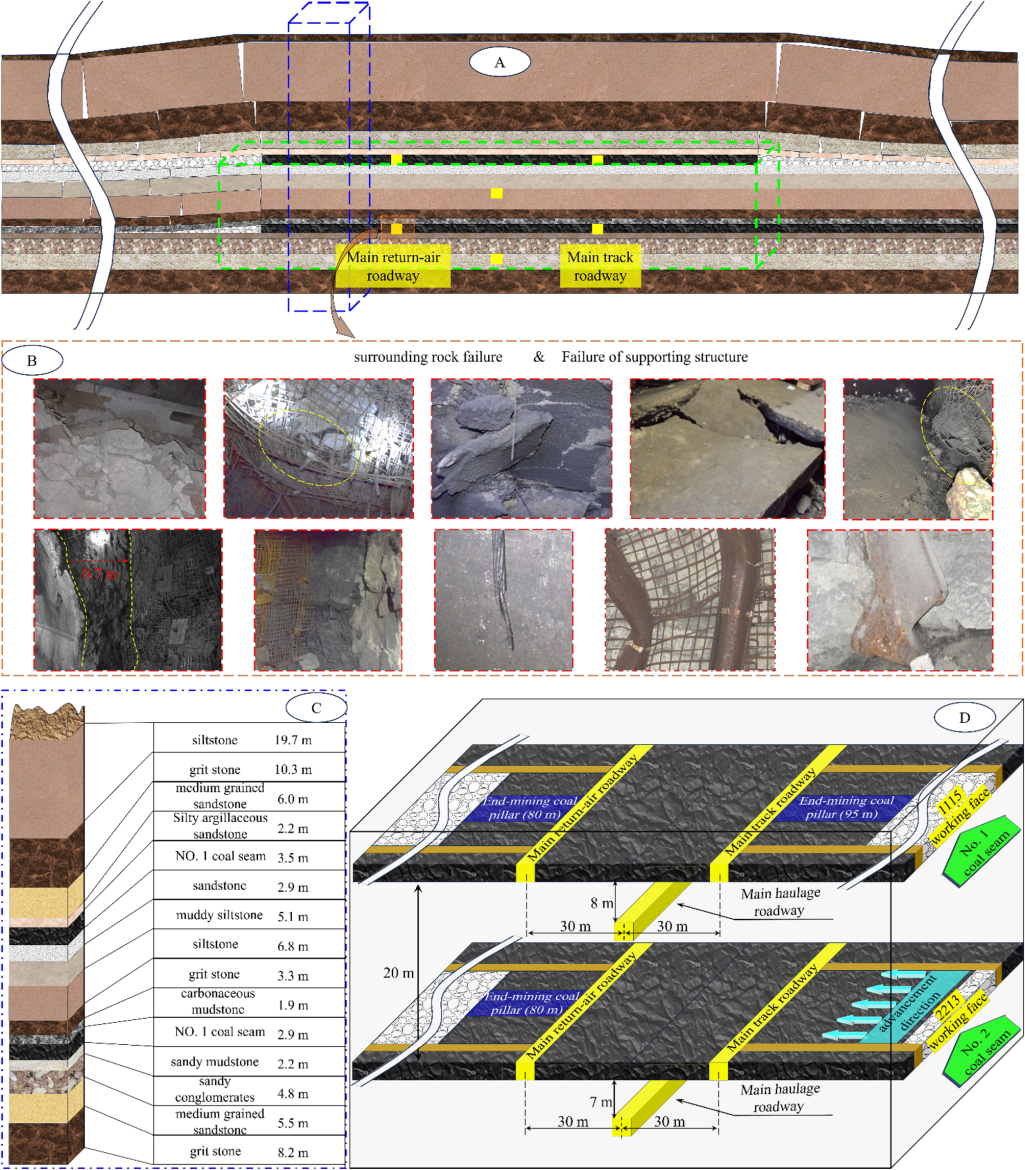
Geological survey of mining area (**A** Mining area structure diagram; **B** Support failure and surrounding rock deformation and failure; **C** Geological histogram; **D** Layout of working faces).

The research project of this paper is located in Datong, Shanxi Province, the depth of the main mining coal seam is about 470 m and the original geostress is 11.75 MPa, which mainly involves two near-level coal seams, No. 1 and No. 2. The average thicknesses of the two seams are 3.5 m (NO. 1) and 3.0 m (NO. 2); the No. 1 seam is mine-out on both sides, leaving 80 m of stop-mining pillars on the left side of the main return-air roadway within the seam, and 95 m of stop-mining pillars on the right side of the main track roadway for the 1115 working face (subsequently referred to as the 1115 WF). The No. 2 coal seam is about 20 m below the No. 1 coal seam, with 80 m of stop-mining pillars on the left side, and the 2213 working face is (the main working face in this study, subsequently referred to as 2213WF) on the right side. The corresponding main roadway of the two seams is horizontally aligned in space; the main haulage roadway of the No. 1 coal seam is 8 m below the coal seam, and the main haulage roadway of the No. 2 coal seam is 7 m below the coal seam. Figure 1 shows the detailed geological conditions of the mining area.

The project aims to maximize the resource recovery rate to ensure safe mining and prevent the waste of coal resources. This means reducing the protective coal pillar’s width, which will undoubtedly complicate the stress superposition of the coal seam. Failure to provide targeted and reasonable support for this unique situation will inevitably lead to unusual deformation and damage in the main roadway, as Fig. 1 shows. Due to multiple superimposed disturbances, the main roadway force is very complicated and uneven. As shown in Fig. 1b, the surrounding rock and support structure of the main return-air roadway are deformed and failed under the influence of superimposed mining, and the failure is obviously asymmetric. Therefore, studying the failure mechanism and development direction of the double-layer isolated island face roadway will help workers design a more reasonable support program to achieve the goal of efficient and safe mining of coal seams.

## Abnormal distribution of stress field under double-layer isolated Island working face

### Loading increase law of abutment pressure of main roadway surrounding rock in double-layer isolated Island face

Under the influence of different mining degrees, the coal rock body of the mining area strata will have a corresponding complex stress redistribution. In this study’s case of the double-layer isolated island face, the stress field is bound to produce serious superposition effects. For this kind of complex stress field environment, using the abutment pressure as an index can more intuitively analyze the stress loading situation of coal seams and the roadway surrounding rock at different stages. Under the excavation step sequence of the actual working conditions in the mining area, to carry out the simulation study: (1) Excavation of the left upper seam → (2) Excavation of the left NO. 2 seam → (3) Excavation of the right NO. 1 seam → (4)–(8) The stop-mining coal pillar of 2213WF respectively take 155 m, 125 m, 95 m, 65 m, and 35 m.

**Figure 2:**
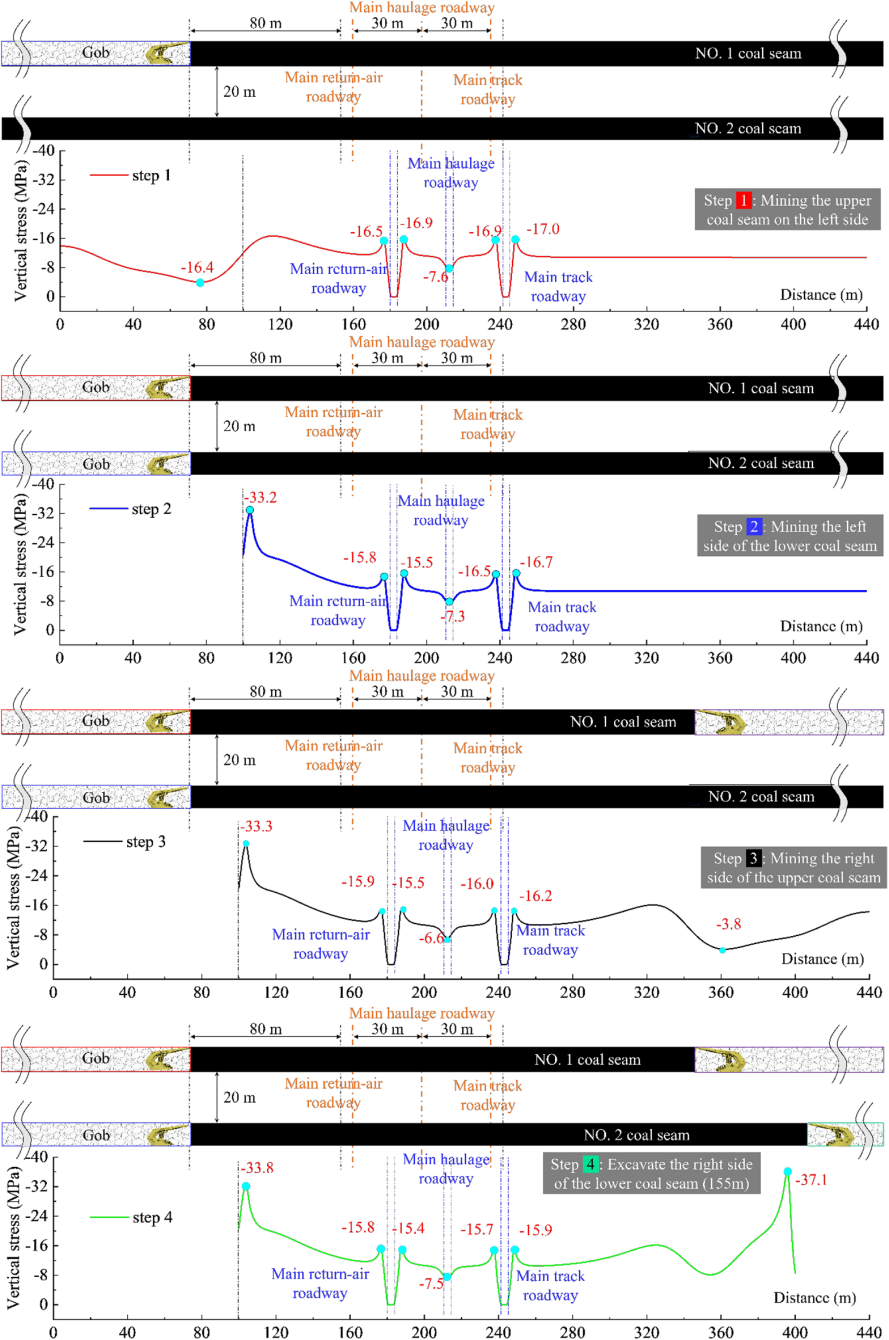
The abutment pressure field of the 2nd coal seam during excavation steps 1 to 4.

**Figure 3:**
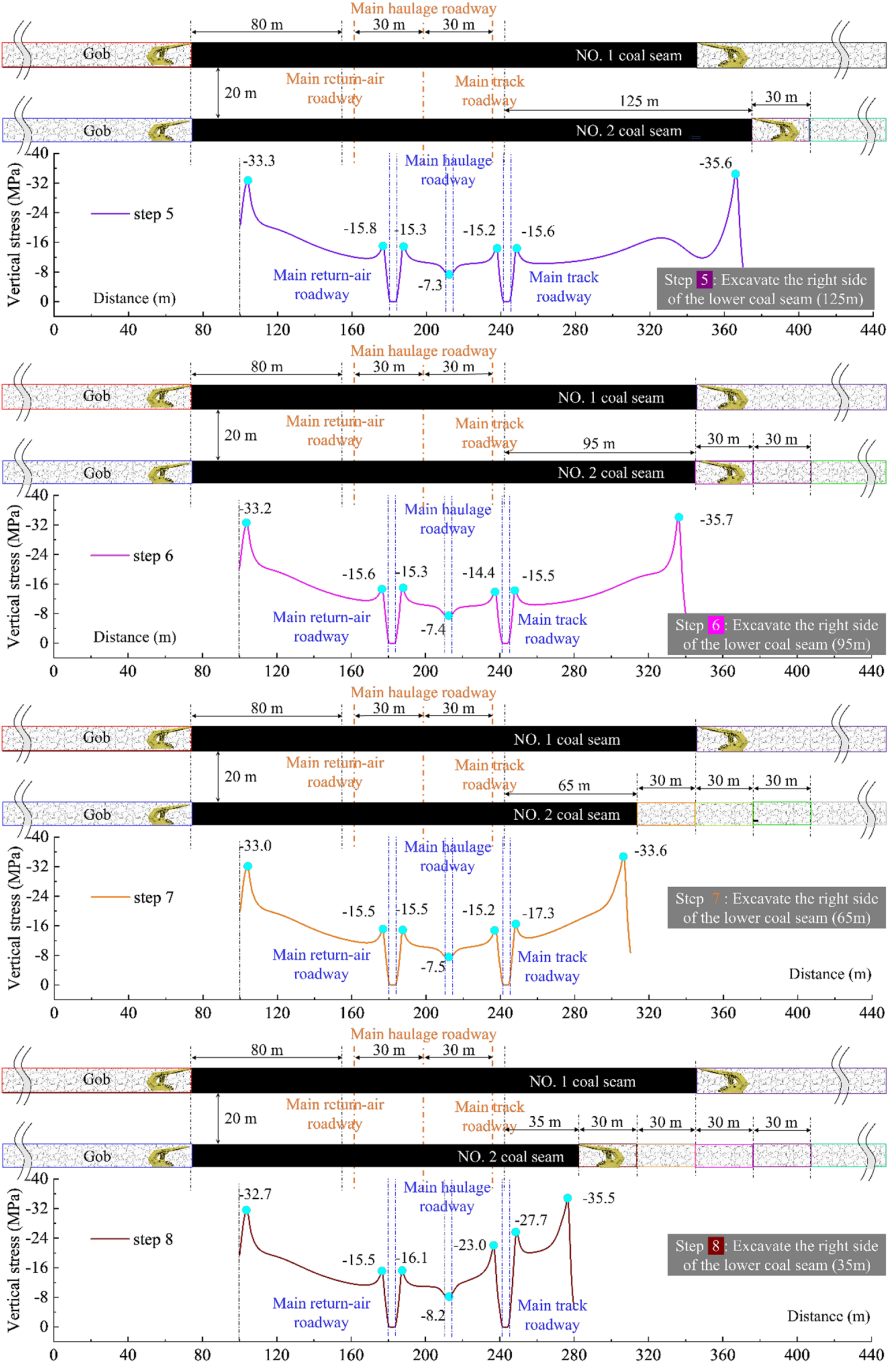
The abutment pressure field of the 2nd coal seam during excavation steps 4 to 8.

**Figure 4:**
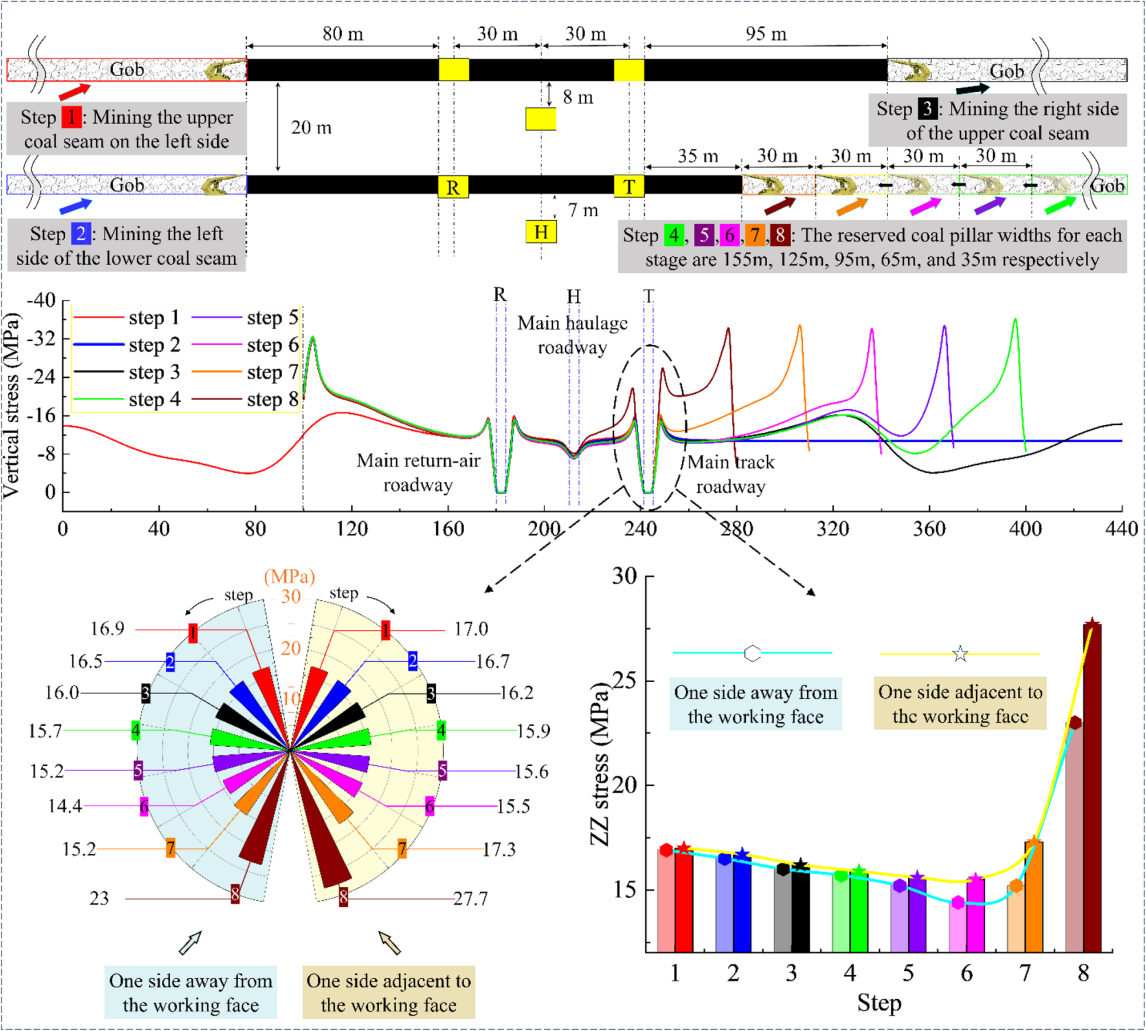
The evolution of No. 2 coal seam’s abutment pressure field of during excavation.

Figures 2, 3, and 4 present the whole process abutment pressure evolution law at the center of the lower coal seam at different mining steps:

1. When excavating only one side (located on the same side of the three main roadways) of the coal body, there is only a slight mining disturbance on the coal seams on the far side.
2. The evolution trend of the peak abutment stress on both sides of the main track roadway is consistent, not a simple linear increase. Before 2213WF advances to coincide with the stop-mining line of the upper coal seam in the horizontal direction, the peak abutment stress at the roadway maintains decreasing (the side adjacent to the working face: 17.0 → 15.5 MPa, and the side adjacent to the island coal pillar: 16.9 → 14.4 MPa); When the 2213WF is ahead of the 1115WF’s stop-mining line, The evolution of abutment stress shows the opposite trend (15.5 → 27.7 MPa on the side adjacent to the working face, 14.4 → 23.0 MPa on the side adjacent to the island coal pillar) and this increasing trend will continue until the seam stop mining.
3. During the whole mining process, under the same pushing distance, the average change rate of the peak abutment stress of the main track roadway was much higher than that of the 2213WF when it was behind the stop-mining line of the 1115WF (0.022 MPa/m). Moreover, with the distance of the 2213WF ahead of the 1115WF (upper) stop-mining line increases, the rate of increase of the peak abutment stress is also faster.

### Superimposed regional loading pattern of principal stresses in the advance working faces of double-layered isolated Island working faces

The principal stresses can centrally reflect the stress situation of a point, making it easy to establish a link between the forces on an object and its destruction. The change of the principal stress direction also reflects the stress field change around the stressed object. Especially in the double-layer isolated island working face condition, analyzing the stress field by the principal stress direction deflection as a comprehensive index is undoubtedly the better choice for researching the destruct development tendency of the roadway and the control direction. Studying the maximum principal stress deflection of the advance working face (2213WF) area of the coal seam under the influence of superimposed mining disturbance can guide setting a more scientific and reasonable coal pillar width for stopping mining.

**Figure 5:**
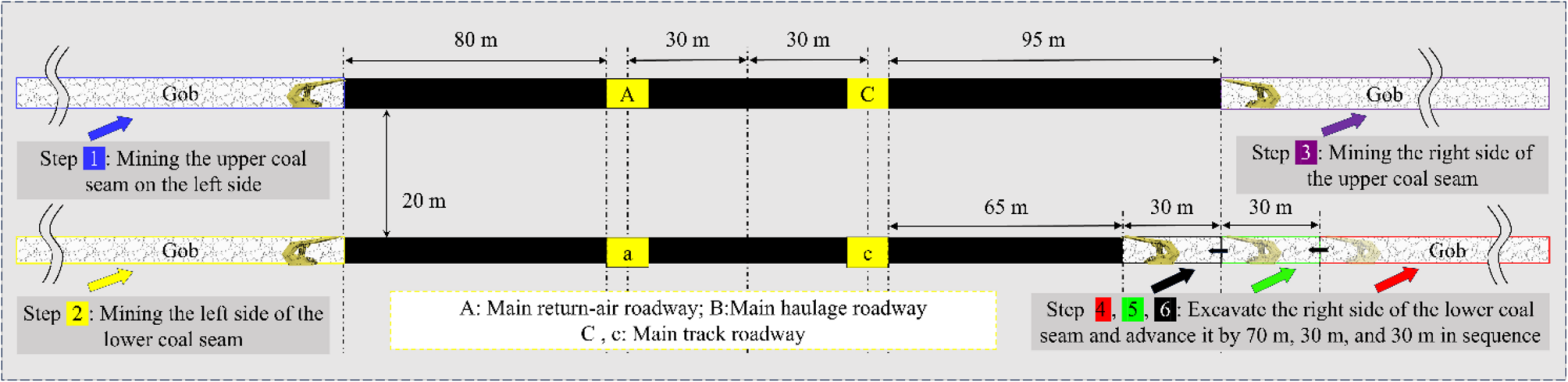
Segmented excavation sequence of studying principal stress.

This paper studied the principal stress direction and its deflection in the advance working face area in the lower seam under different excavation steps during the whole process of mining from the initial excavation to the stop mining of the 2213WF (Fig. 5 shows the advancement steps involved in the study and the arrangement of the measurement points). In the NO. 2 coal seam, the measuring points were set 20 m to the right of the stop-mining line of the left side working face and every 20 m to the right horizontally, and the measuring points were located on the centerline. The principal stress direction was calculated from the numerical calculation of the stress components at the measuring points, comparing and analyzing the deflection situation.

**Figure 6:**
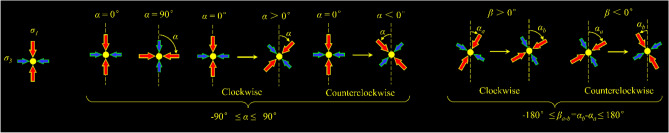
The setting of maximum principal stress direction angle: *α* and *β*.

For convenient comparative analysis, the deflection of the maximum principal stresses is represented by the values of α and β as specified in Fig. 6.

**Figure 7:**
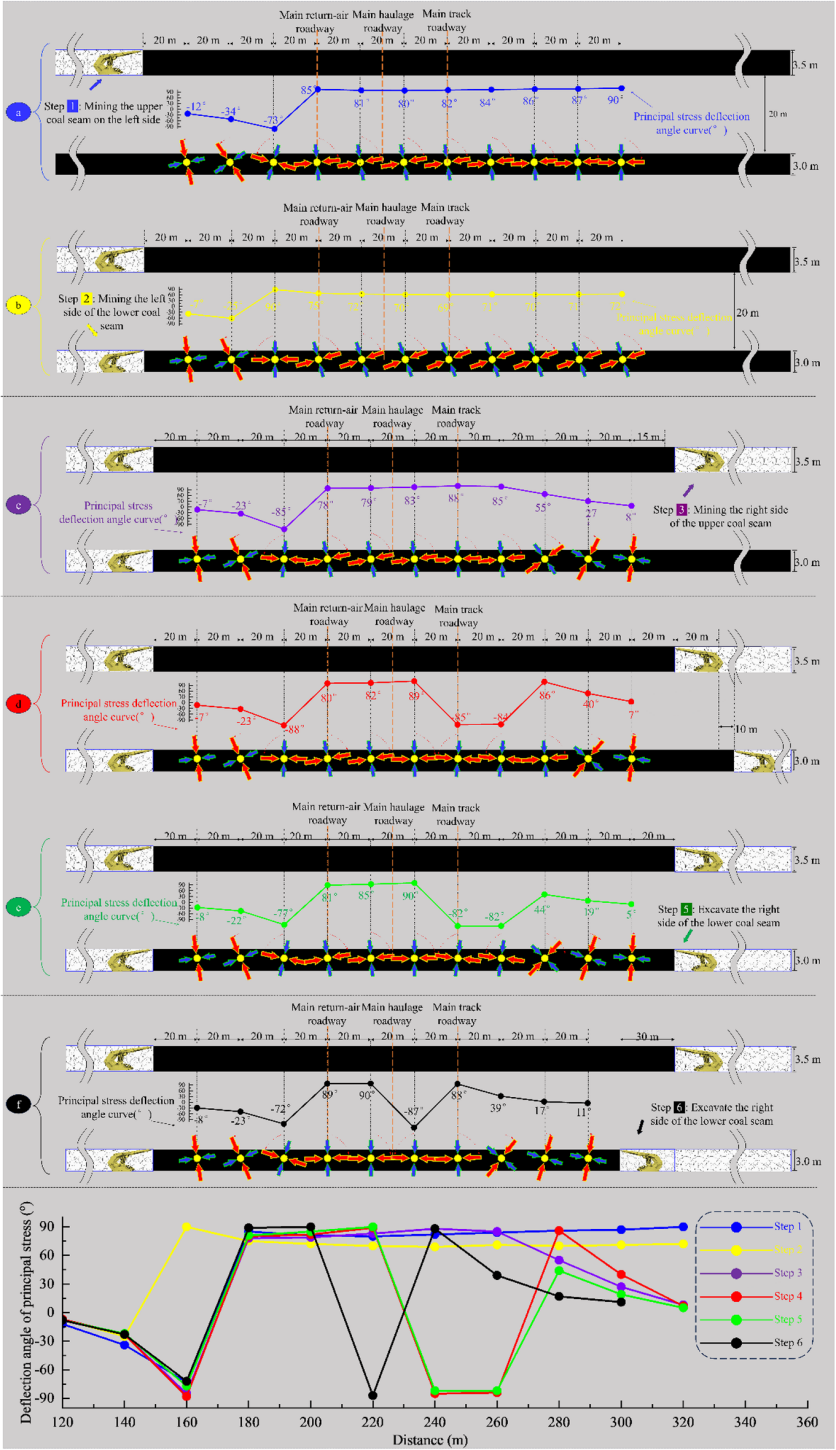
The evolution of No. 2 coal seam *α* in the whole mining process.

**Figure 8:**
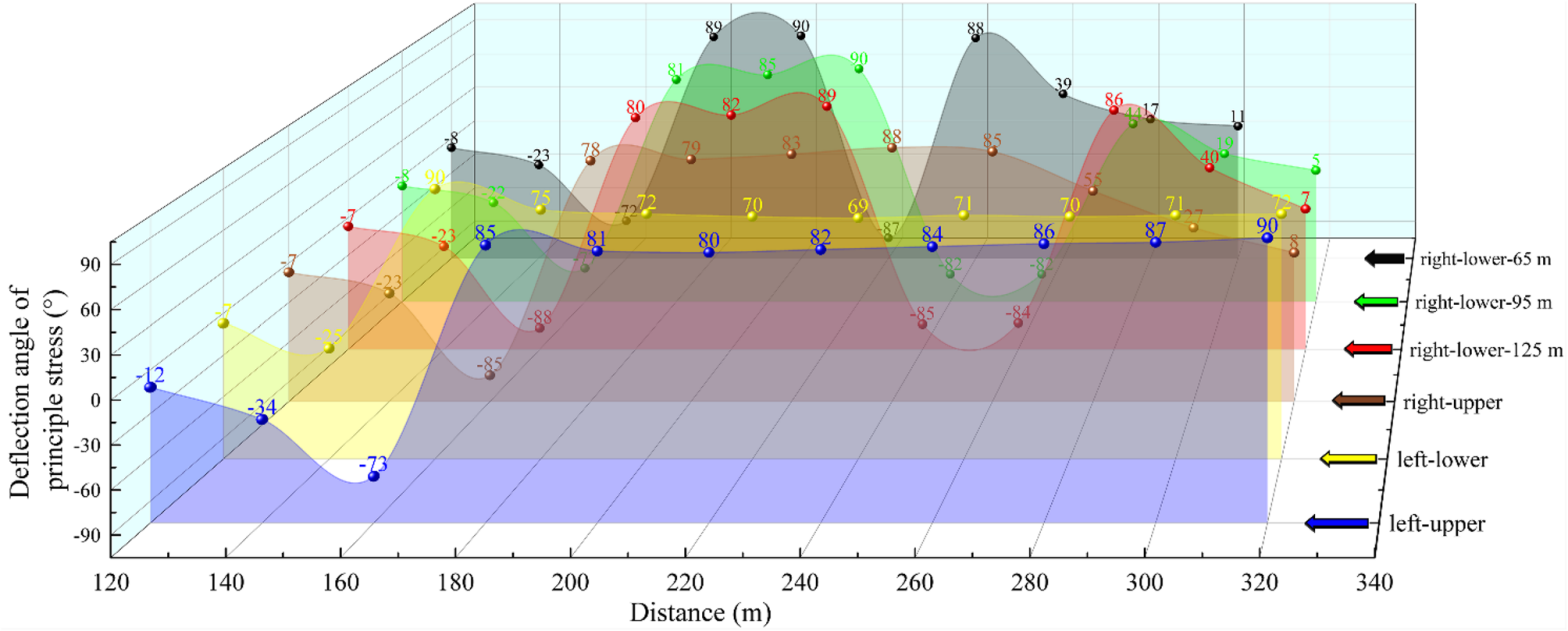
The comparison of the No. 2 coal seam’s *α* throughout the entire mining process.

**Figure 9:**
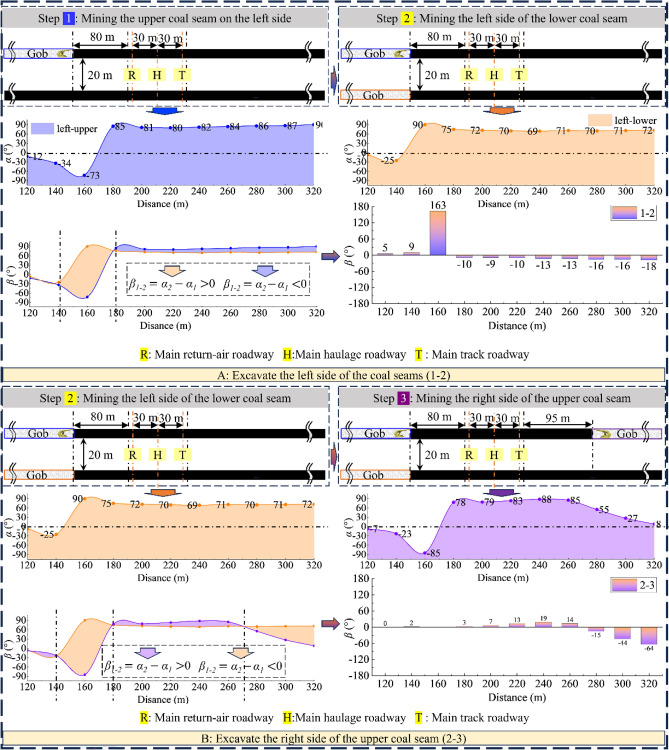
Comparison of *α* and *β* between adjacent excavation steps (excavation steps 1 to 3).

**Figure 10:**
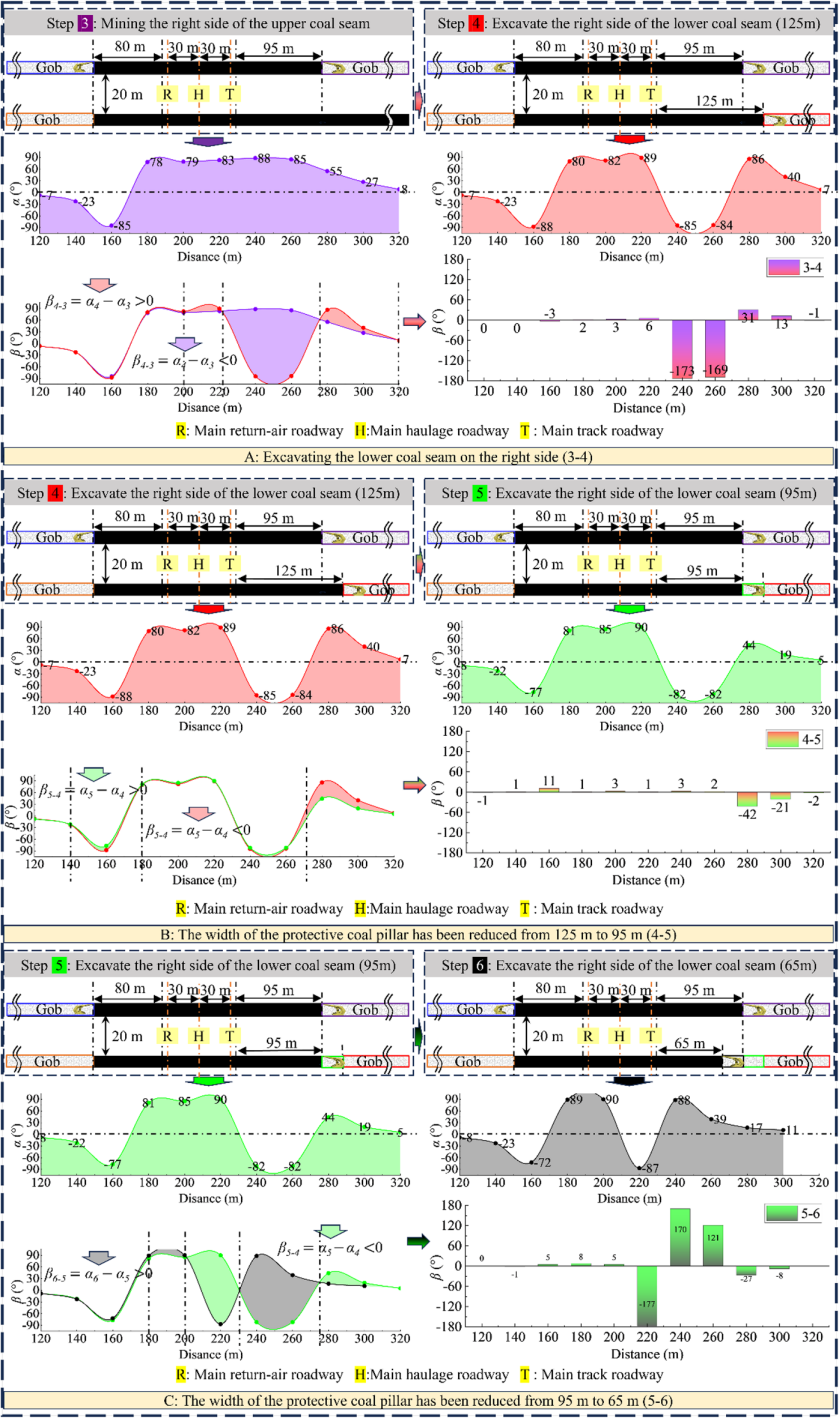
Comparison of *α* and *β* between adjacent excavation steps (excavation steps 3 to 6).

As Figs. 7, 8, 9 and 10 show, the value of α at each measurement point is significantly under the mining disturbance, and the values of *α* and *β* show a typical nonlinear evolution under the effect of multiple superpositions of the influence of mining in each advancing step.

1. The mining of the No. 1 coal seam makes the *α* value ahead of the working face area, show an apparent asymmetric loading phenomenon: the maximum principal stress in the area of 40 ~ 60 m ahead of the working face is deflected to the gob side. The α value gradually increases from the vicinity to the distance, and the *α* outside the area of 60 m shows a direction of deflection away from the gob.
2. The mining of the No. 2 coal seam makes the distance from the location where the *α* value ahead of the working face deflects from “ − ” to “ + ” to the working face show an apparent reduction, from 40–60 m to 40 m. The *α* value at the location far away from the working face decreases, and the phenomenon of deflected loading intensifies.
3. The mining of the 1115WF makes the loading pattern of *α* value in the nearer area (40 ~ 60 m) ahead of the 1115WF of the No. 2 coal seam similar to that when mining the left side of the isolated island face, however, under the influence of the isolated island face on the left side mining, the *α* value in the farther area does not show the “ + ”,“ − ” conversion phenomenon.
4. With the increase of advancement distance of 2213WF and the gradual decrease of coal pillar width, the *α* value in the range of 60 m ahead of the 2213WF shows a nonlinear loading trend of decreasing and then increasing, which corresponds to the spatial relationship between 2213 and 1115WF: lagging → (*α* decreasing) → overlapping-(*α* increasing) → aheading. The *α* value outside of 60 m gradually increases with the mining.

During the whole process of mining coal seams on both sides of the double-layer island face, the maximum principal stress shows the tendency of favoring the gob when near the working face and favoring the coal body at the place adjacent to the island coal pillar.

### Deflection of the maximum principal stress at the main roadway by superimposed disturbances

**Figure 11:**
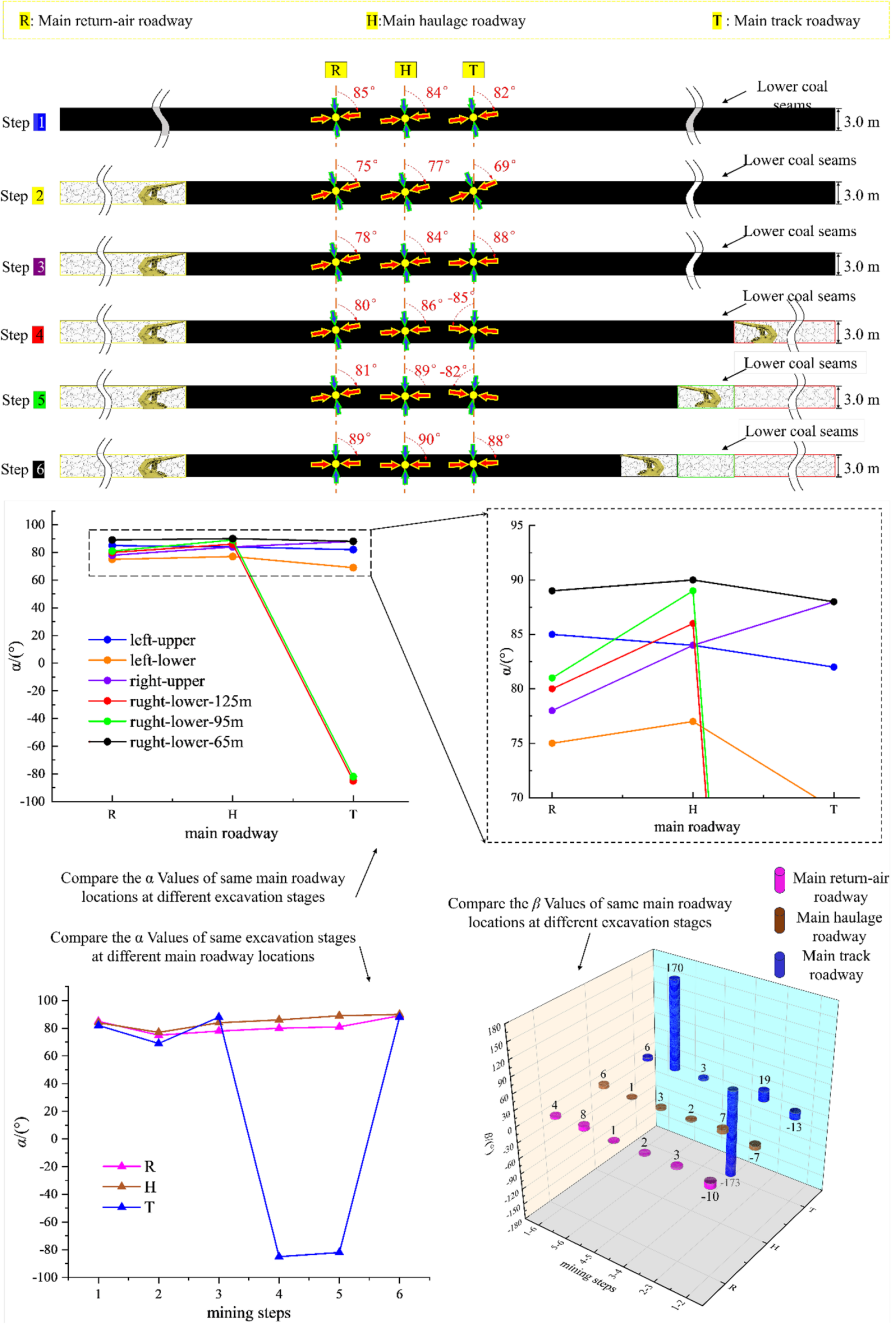
The *α* and *β* in the location of the main roadways evolution during the entire excavation process.

Figure 11 compares the *α* and *β* at the coal seams corresponding to the three main roadways (a: comparing the *α* value of the same main roadway under different advancing steps; b: comparing the α value at the perimeter rock of different main roadway under the same mining steps; and c: comparing the *β* value at the perimeter rock of the main roadway after experiencing different mining steps), and makes a pertinent analysis for the main roadway disturbed under the disturbing of the double-layer and double-side mining.

After comparative analyses of the three types the figure above shows, it is clear that:

1. Under the condition of double-layer island working face, the main track roadway (adjacent to 2213WF) is most intensely disturbed by superposition. The change of *α* and *β* of the main track roadway in the main roadway group is the largest under the same push-forward distance.
2. The times of the “backtracking” phenomenon of the *β* are different in different main roadways during the mining process. The *β* at the main return-air roadway only appeared once (when 1115WF mining, the *β*-value appeared “ − ” → “ + ”) and lasted until the 2213WF stop-mining.

Moreover, after excavating the 1115WF, the *β* value at the main track roadway shifted from “ − ” → “ + .” With the mining of the 2213WF, the *β* value at the main track roadway appeared to have a deflection phenomenon many times (“ + ” → “ − ” → “ + ”), which is very different from the symmetrical spatial and geologic relationship of the two main roadways. Therefore, studying the change law of the *α* and *β* in the main roadway under such geological conditions is of great significance for predicting the direction of the surrounding rock damage and reasonable control.

### The law of deviatoric stress regional loading and failure modes on the main roadway surrounding rock of the double-layer Island working face

In the analysis of elastic–plastic mechanics^26,27^, the deviatoric stress integrally reflects the changes of principal stresses and dominates the plastic deformation of the object. The main roadway surrounding rock will inevitably appear with asymmetric plastic deformation or even damage with the superimposed-mining impacts. To truly grasp the roadway deformation and destruction of the law and development trend, using deviatoric stress, which controls the object’s plastic deformation as an indicator, combined the *α* of the surrounding rock under the corresponding advancing steps, systematically analyzed the deviatoric stress field situation of advance 2213WF area in the advancing, in order to complete the prediction of roadway possible damage direction and the determine of surrounding rock scientific control method.

**Figure 12:**
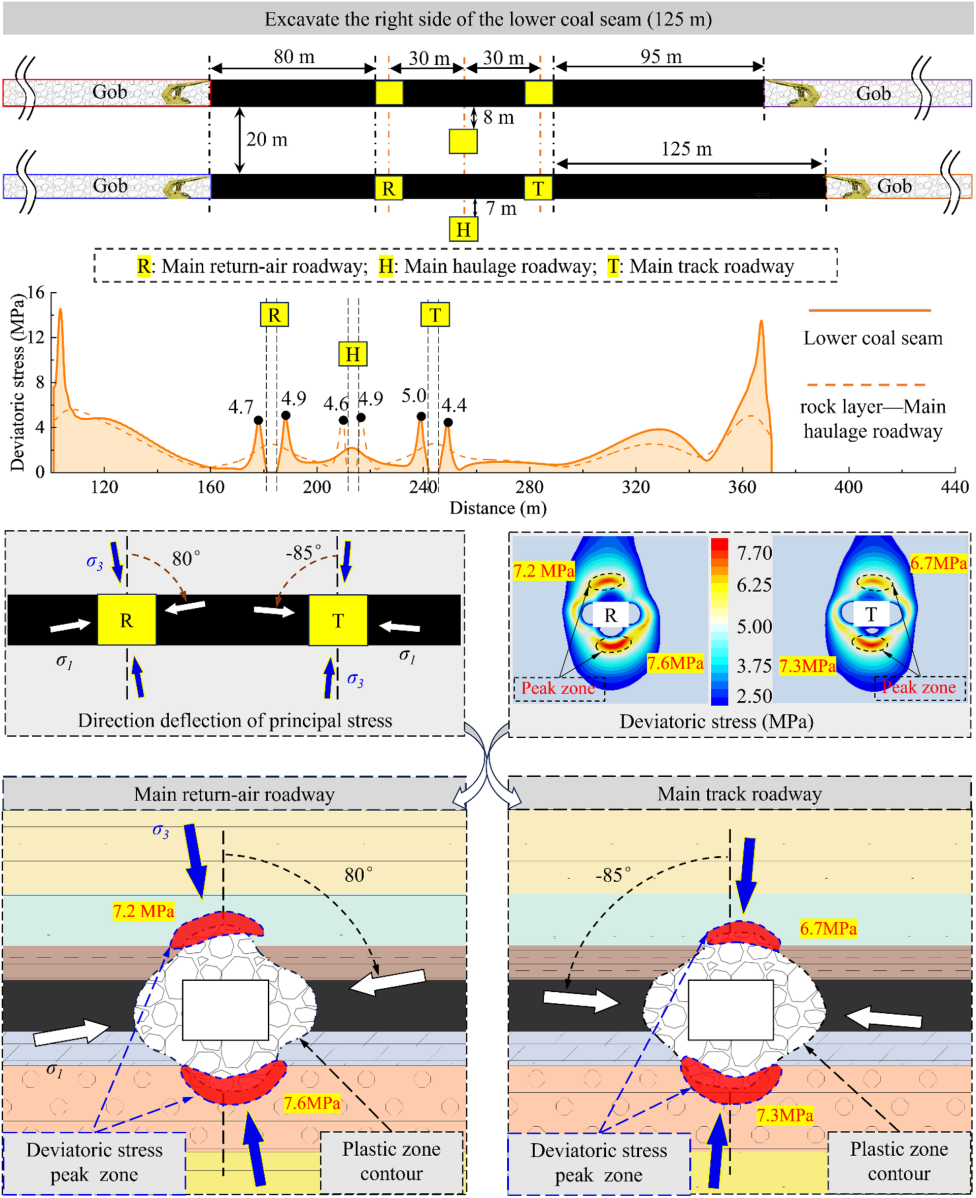
The evolution of deviatoric stress in the surrounding rock of the main roadway when the width of the coal pillar is 125 m.

**Figure 13:**
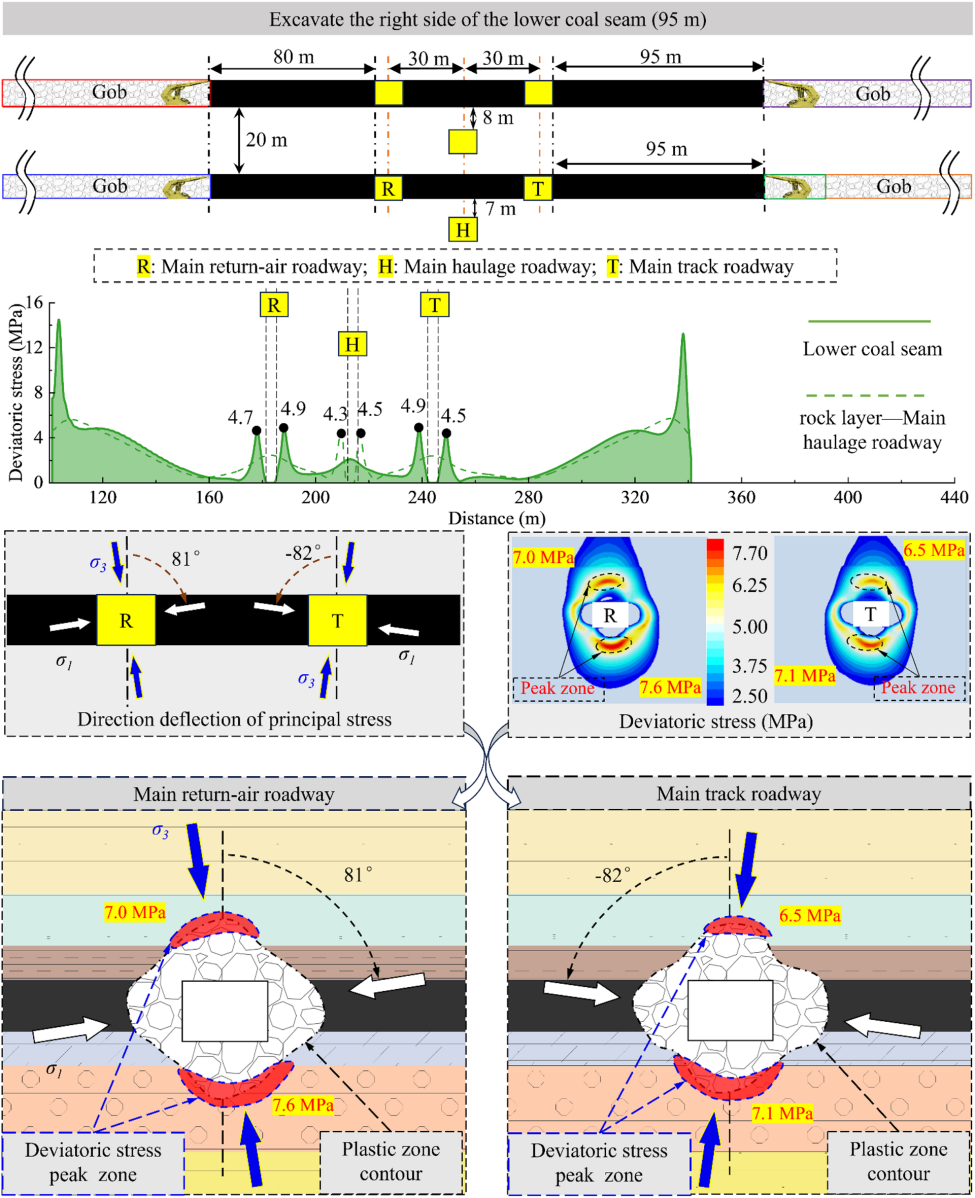
The evolution of deviatoric stress in the surrounding rock of the main roadway when the width of the coal pillar is 95 m.

**Figure 14:**
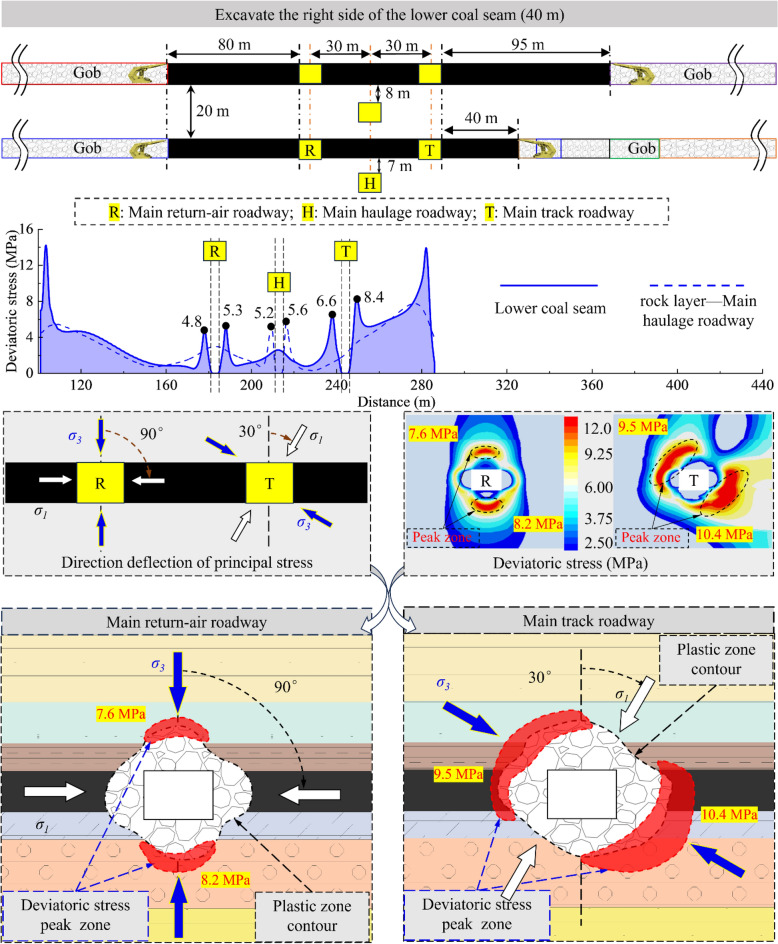
The evolution of deviatoric stress in the surrounding rock of the main roadway when the width of the coal pillar is 40 m.

**Figure 15:**
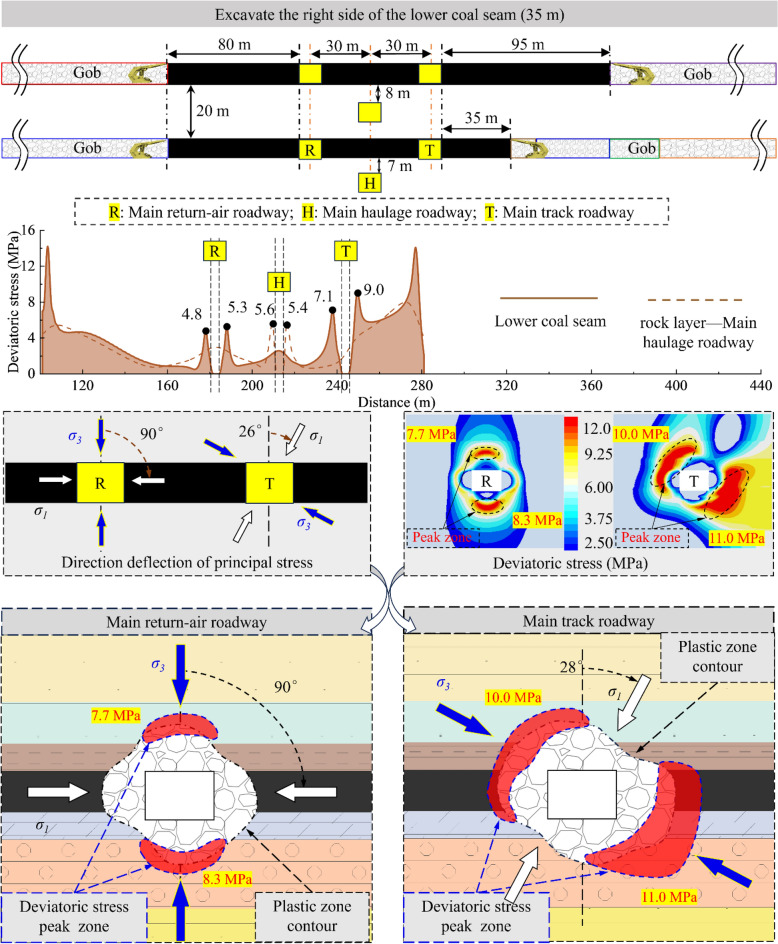
The evolution of deviatoric stress in the surrounding rock of the main roadway when the width of the coal pillar is 35 m.

According to the analysis results, the sensitivity of the surrounding rock to the superimposed mining effect will gradually decrease due to the increase in the distance from the 2213WF. For the main roadway far away from the mining side, the deviatoric stress has no noticeable change during the mining period of 2213WF. Therefore, this paper mainly studies the development of surrounding rock deviatoric stress in the main roadway (main track roadway) close to the 2213WF. Figures 12, 13, 14 and 15 show:

1. The peak deviatoric stress zone (subsequently abbreviated as PDSZ) evolution on the main roadway two ribs is generally consistent with the abutment pressure. The horizon relation between the 2213WF and the 1115WF stop-mining line determines the trend of the PDSZ evolution of the roadway at this stage: the PDSZ at the surrounding rock continues to decrease when the 2213WF is lagging behind the 1115WF, and the opposite is the case when it is advance 1115WF.
2. The PDSZ on the roadway show typical asymmetry. The high stress of the PDSZ in the rib part of the main track roadway adjacent to the 2213WF is smaller than the high stress in the rib part far away from the 2213WF when the 2213WF is behind the 1115WF stop-mining line; it is the opposite when it is ahead of the 1115WF stop-mining line. When the coal pillar width in the main track roadway is from ∞ m → 125 m → 95 m → 40 m → 35 m, the peak deviatoric stress is from 7.3 MPa → 7.1 MPa → 10.4 MPa → 11.0 MPa; the maximum stress loading factor is *L* = 2.1. The main track roadway peak deviatoric stress is 4.82 MPa → 4.60 M Pa → 6.99 MPa, and the *L* is 1.45.
3. As the distance of 2213WF exceeding 1115WF increases gradually, the deflection phenomenon of the PDSZ by the superposition effect becomes more and more obvious, and the peak stress in the core zone increases along with the increase of the volume of the PDSZ.
4. There is a correspondence between the deflection of the PDSZ and the change of the *α* in the corresponding area, and the line of the minimum principal stress direction runs through the PDSZ, pointing to the direction of the development of the PDSZ at this time. Clarifying the development pattern and evolution trend of the PDSZ of the main roadway is meaningful to the early warning and control of the damage location.
5. When the main roadway coal pillar’s width is reduced from 40 to 35m, the PDSZ shows a trend from pointing to two sides to pointing to roof and floor, combined with site engineering conditions, considering the output and safety factors, the protective coal pillar width is determined to be 40m.

## Evolutionary patterns and control principle of the main roadway surrounding rock with double-layer isolated Island working face

### Development patterns of the PDSZ of surrounding rock

**Figure 16:**
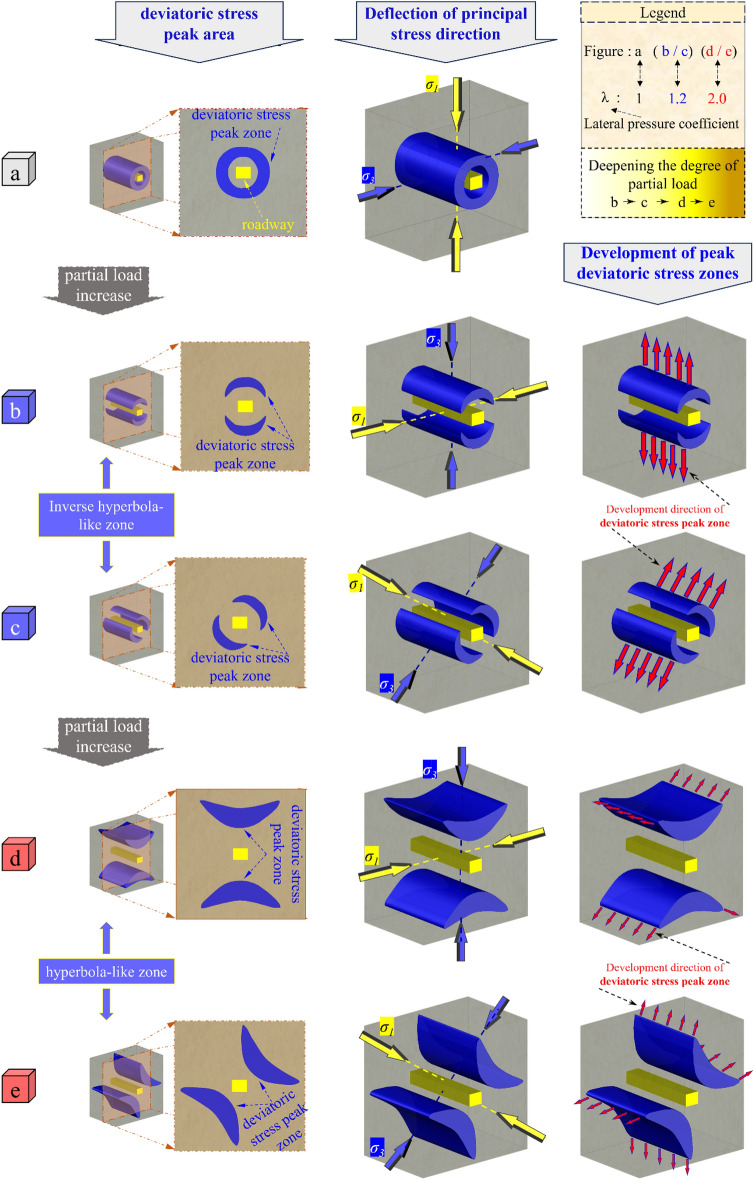
Evolution model of deviatoric stress and the critical control direction of surrounding rock under different stress local loading.

The principle stress deflects at different degrees, and the shape of the PDSZ which can reflect the change of the *α* and the evolution mode are also different. The different evolution patterns of the PDSZ dominate the roadway deformation and damage in different stress-superimposed loading conditions. Therefore, to achieve the absolute reasonable roadway deformation control, according to the different development stages of the PDSZ, determine the corresponding scientific support methods. According to the above-mentioned evolution law of the roadway PDSZ in the double-layer island working face main roadway, combined with the change of the *α*, we get the evolution pattern of the PDSZ (the critical zone of the surrounding rock control) under various types of deviatoric loading conditions, as in Fig. 16.

The development morphology of the roadway PDSZ under the mining disturbance is roughly classified into three categories: (1) round tube body (the surrounding rock is in an undisturbed isobaric environment). (2) Anti-class hyperbolic body (stress loading leads to a change of *α*, and the morphology of the PDSZ begins to evolve along with the deflection of the minimum principal stress). (3) Class hyperbolic body (the deflection loading of the stress region is aggravated, and the change of *α* is increased so that the PDSZ is developed further towards the depth surrounding rock in multiple directions).

According to the spatial location and evolution trend of the PDSZ of the roadway surrounding rock, there are nine evolution modes. Among them, the round tube body like PDSZ shows a single evolution mode, and the anti-class hyperbolic body and the class hyperbolic body type PDSZ each has four evolution modes respectively, which are along the direction of the roof-floor plate, along the direction of the two ribs of the roadway, along the left rib-roof transition zone of the roadway, and the right rib-roof transition of the roadway zone.

Based on the actual development pattern and evolution mode of the PDSZ under the actual working condition, the asymmetric critical zone cooperative control method of this paper adopts the highly targeted high-strength prestressing anchor cable structure to strengthen the support for the roadway possible destructive direction.

### Novel asymmetric critical zone collaborative control method based on the PDSZ

Considering the resource recovery rate and safety factors, the main track roadway coal pillar is 40 m. Under the superposition of stresses in such a stratigraphic relationship, the roadway PDSZ shows the development morphology of anti-class hyperbolic body, and evolves along the transition zone of the left rib-roof of the roadway.

**Figure 17:**
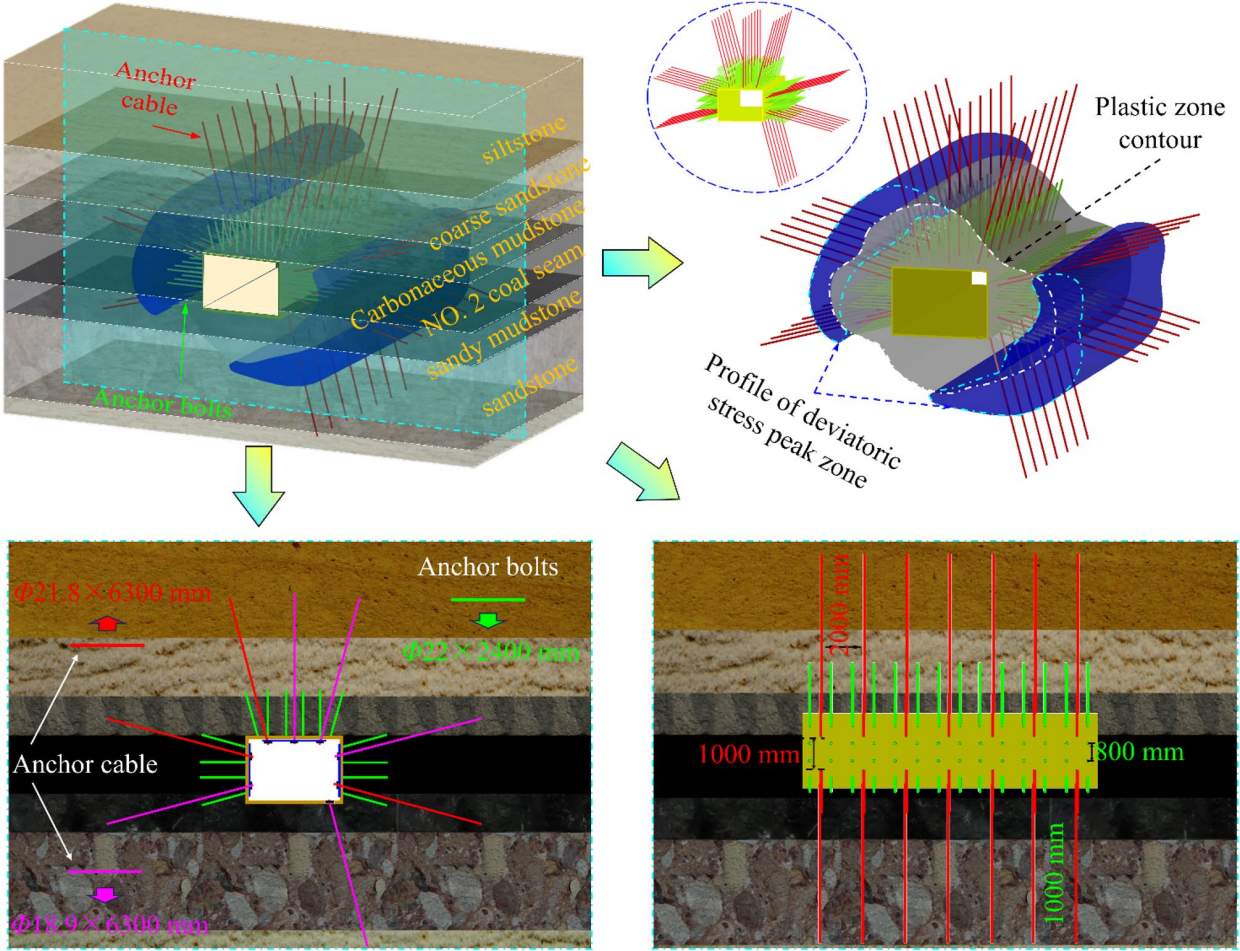
Supporting scheme and principle of the main track roadway.

Based on the synergistic control method of the asymmetric critical zone of the surrounding rock, propose a targeted support program for the trackway in the lower coal seam. As Fig. 17 shows, three anchor cables with specifications of Φ18.9 × 6300 mm and spacing of 1500 × 2000 mm (horizontal direction × axial direction of the roadway) are arranged on the roof. Two reinforcing anchor cables are installed in each of the two ribs of the roadway; the upper anchor cable specifications of the left rib and the lower anchor cable of the right rib are Φ21.8 × 6300 mm, and the rest are Φ18.9 × 6300 mm. The anchor cables near the roof and floor plate have an angle of 15° to the horizontal direction, and each side of the anchor cables is connected by channel steel: the spacing between the two ribs of anchor cables is 1600 × 2000 mm (vertical direction × axial direction of the roadway). The pallet specification is 300 × 300 × 16 mm (length × width × thickness); the anchor bolts adopt Φ22 × 2400 mm left-handed non-longitudinal reinforcement rebar anchor bolts, with the inter-row spacing of 800 × 1000 mm, and the pallets adopt 150 × 150 × 10 mm (length × width × thickness) disc-shaped pallets. The thickness of slurry sprayed on the two ribs and the roof plate is 100 mm, and the strength grade is C20. The floor plate of the roadway section is equipped with a single anchor cable of Φ21.8 × 6300 mm, which is close to the stop-mining coal pillar side of the main roadway, 20 cm away from the gang, and at an angle of 15° to the vertical direction. In order not to hinder the normal functioning of the main track roadway, a groove with a depth of 10 cm was constructed in the floor to embed the anchor cable and its pallet in it.

**Figure 18:**
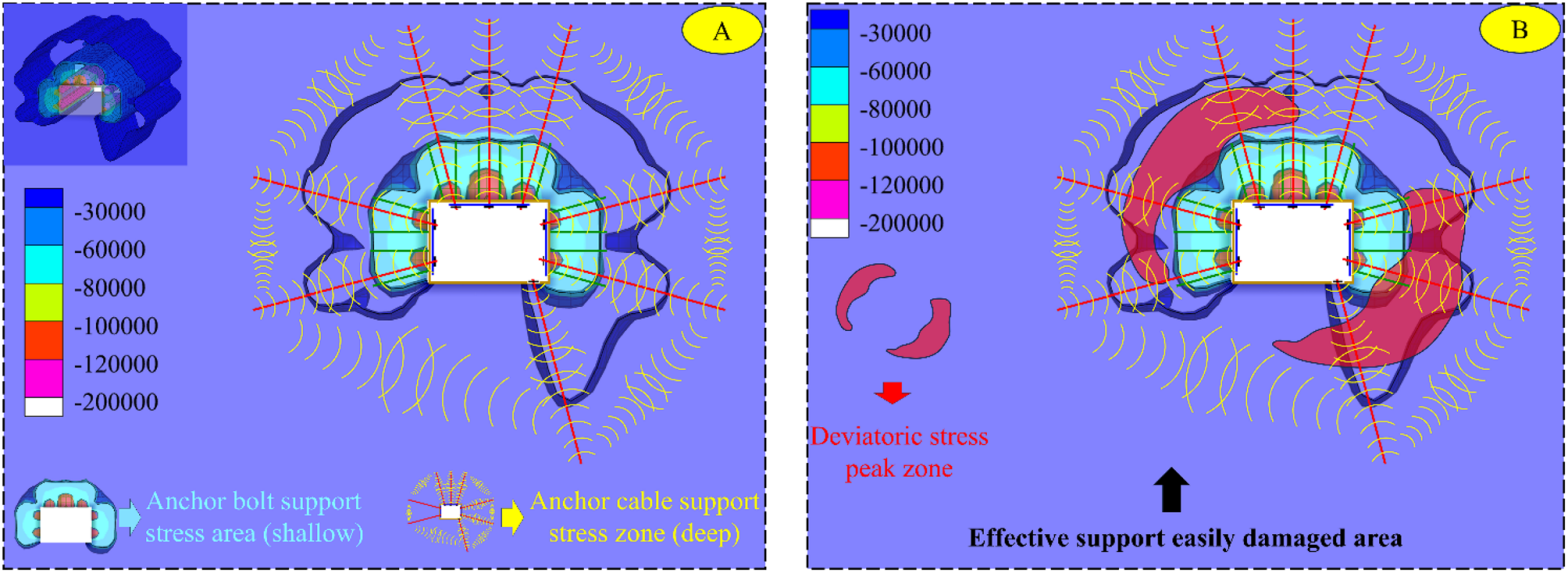
Stress field of supporting structure (**A**: Stress field network of supporting systemd; **B**: Effective support schematic).

The conventional gang anchors can avoid the shedding of the broken perimeter rock on the surface of the roadway, but it cannot play an effective support role for the critical zone (the PDSZ) where the roadway deformation damage is developed. As Fig. 18 shows, after adopting asymmetric critical zone directional synergistic support, compared with the conventional support method, there still exists a deep compressive stress zone formed by the reinforcing anchors. The directional reinforcing anchor cables of the two ribs form two groups of effective deep compressive stress areas in the critical area of PDSZ, and synergize with the anchor cables of the roof plate to form a systematic reinforcing stress field network. This achieves the effective coverage of the perimeter rock bias load key area, accomplishes the effective transmission of the shallow mining power of the perimeter rock to the deep elastic rock body, and then achieves the purpose of controlling the damage of the roadway.

## On-site monitoring

**Figure 19:**
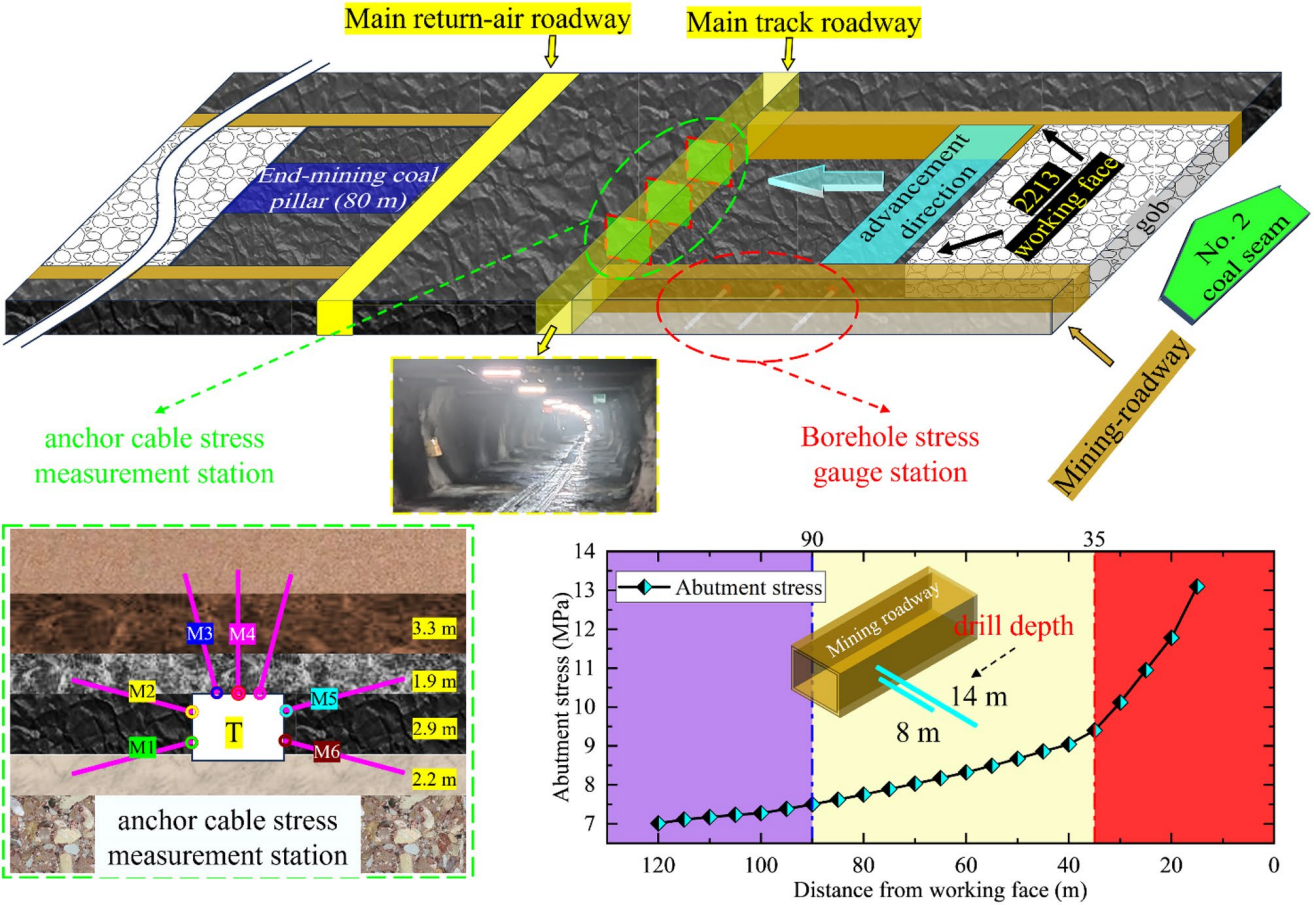
On-site monitoring principle and abutment pressure of mining roadway.

Working face mining roadway in the direction of their respective axes to arrange the measurement station by fitting the monitoring results to analyze the roadway’s response to mining pressure. The interval of each station in the roadway is 10 m, and the observation contents are as follows: (1) the mining-roadway abutment pressure; (2) the force of the anchor cable in the section of the main track roadway. By comprehensively analyzing the changing trend of the above indexes, the change rule of the stress field of the roadway is clarified when the 2213WF stop-mining pillar width is different to determine the reasonability of the stop-mining pillar width of 2213WF. Figure 18 shows the monitoring principle, and the drilling stress gauge measurement points are laid in the mining-roadway of the 2213WF of the lower coal seam. Drill holes penetrate 14 m into the coal, and the abutment stress monitoring results in the roadway are as in Fig. 19.

During the advancement of the 2213WF: when the distance between the stress gauges is greater than 90 m, the stress gauge fluctuation readings is not obvious, and the overall situation is stable; when the distance between the stress gauges is between 90 and 35 m, the stress gauges values begin to increase slightly; when the distance between the stress gauges is less than 35 m, the values begin to increase sharply in comparison with the previous stage. As the 2213WF advances, the high-sensitivity borehole stress gauges in the station gradually approach, and the growth rate of the supporting stress value in the advance 2213WF area increases significantly.

**Figure 20:**
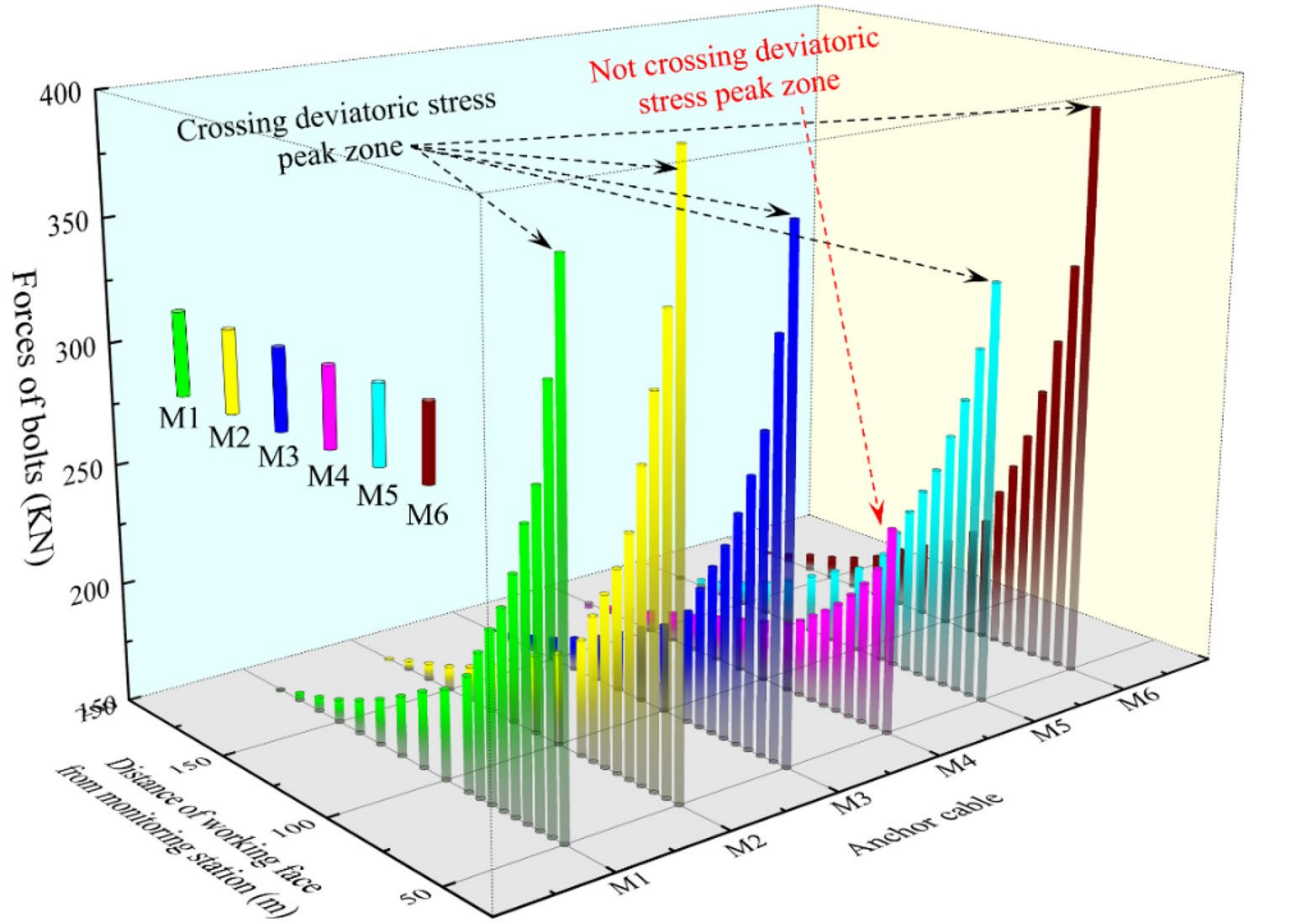
Stress situation of anchor cables at the cross-section of the main track tunnels.

The monitoring of anchor cable support resistance can reflect in real time the influence range of the working face mining and the roadway deformation state to guide the determination of the reasonable stop-mining coal pillar width of the 2213WF. Figure 19 shows the arrangement of measuring stations in the roadway, and Fig. 20 shows the monitoring results.

With the advancement of the 2213WF, the overall trend of the stress gauge readings of the anchor cables in the No. 2 coal seam main track roadway coincides with the trend of developing the PDSZ. 2213WF after leaving 40 m of stop-mining pillars, the stress gauge readings of the anchor cables that pass through the main roadway PDSZ are higher than the other zones, which is the same direction as that of the roadway damage, and shows the typical asymmetric characteristics. Comparison of M1, M2, M3, M4, M5, and M6 shows that the anchor cable stress gauge readings in the main roadway PDSZ are the most sensitive to the effect of mining. The roadway PDSZ is reasonably the critical zone of the roadway control. The on-site monitoring results coincide with the critical zone of main roadway deviatoric stress regional loading in the above study, which shows that the theory of regional stress loading of the surrounding rock is reasonable.

## Conclusion

This paper adopts numerical simulation field monitoring and other methods to explore the surrounding rock failure mode of the double-layer isolated island face main roadway under different degrees of regional stress loading conditions. Conclusions were obtained as follows:

1. When the 2213WF is behind the stop-mining line of the NO. 1 coal seam, the side near the 2213WF of the surrounding rock, the peak stress is smaller than the side away from the 2213WF, and the opposite is the case when advance. The reversal of the main stress direction deviation before and after pushing the upper stop mining line on the working face is consistent with the reverse loading trend of the surrounding rock stress. (the principle stress direction ahead of the 2213WF is: away from the 2213WF → towards the direction of the 2213WF slewing → away from the 2213WF).
2. The roadway is affected by the superposition of multiple mining, and three possible development patterns of the peak deviatoric stress zone and nine corresponding evolution modes (one round tube body type, four anti-class hyperbolic body types, and four hyperbolic body types) will appear. Under the isobaric environment without regional stress loading, the PDSZ of the surrounding rock is round tube-like, the surrounding rock is stable. When the degree of regional stress loading is weaker, the PDSZ is in the shape of the anti-class hyperbolic body and will develop along the two ends of the line of the minimum principal stress direction at this time, and the deformation of the roadway is relatively controllable; when the degree of regional stress loading is deepened, the PDSZ is in the form of class hyperbolic body type, potential deformation and damage direction are doubled, making it difficult to form effective support for it.
3. 2213WF left 40 m protect coal pillar, the roadway PDSZ was developed along the transition zone of the left rib-roof in the form of anti-class hyperbolic body development. After targeted support through the asymmetric critical zone synergistic control method, on-site monitoring shows that the anchor cable stress gauge reading in the peak partial stress area is higher, the roadway deformation and failure is controllable, and the support program is reasonable and effective.

## Data Availability

All data generated or analysed during this study are included in this published article.
